# 4-Bromo-*N*,*N*′-di­phenyl­benzimidamide *N*′-oxide

**DOI:** 10.1107/S2414314624009684

**Published:** 2024-10-08

**Authors:** Arindam Saha, Daniel Chartrand, Mihaela Cibian, Thierry Maris, Garry S. Hanan

**Affiliations:** a Département de Chimie, Université de Montréal, Complexe des sciences, 1375, Avenue Thérèse-Lavoie-Roux, Montréal, Québec H2V 0B3, Canada; b Département de biochimie, chimie, physique et science forensique, Université du Québec à Trois-Rivières. 3351, boul. des Forges, CP 500, Trois-Rivières, Québec, G9A 5H7, Canada; University of Aberdeen, United Kingdom

**Keywords:** amidine *N*-oxides, ligand synthesis, crystal structure

## Abstract

The title compound, C_19_H_15_BrN_2_O, crystallizes with two similar mol­ecules in the asymmetric unit. The extended structure features dimers linked by pairs of N—H⋯O and C—H⋯O hydrogen bonds. The HNCNO moiety of the title compound shows delocalization over the N—C—N part, as evidenced by the similar C—N bond distances.

## Abstract 2

Bright-yellow single crystals of the title compound were synthesized as part of a project that explores the formation of earth-abundant transition-metal complexes of amidine oxides (AMOXs), to study their photophysical and redox properties. The HNCNO moiety of the compound shows double-bond delocalization over the N—C—N part, evident from the similar C—N bond distances.

## Graphical abstract

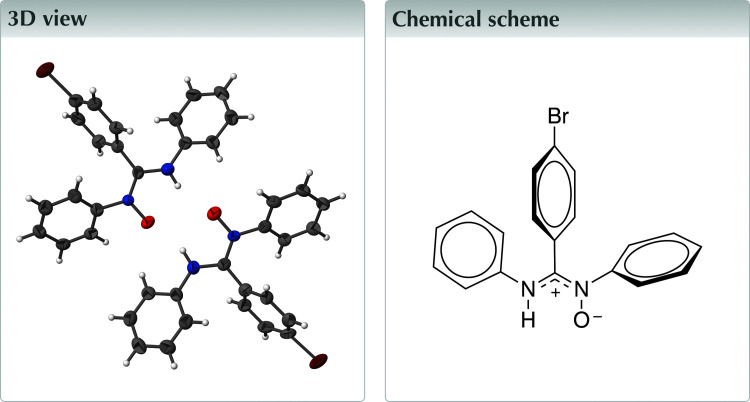

## Structure description

**Figure 1:**
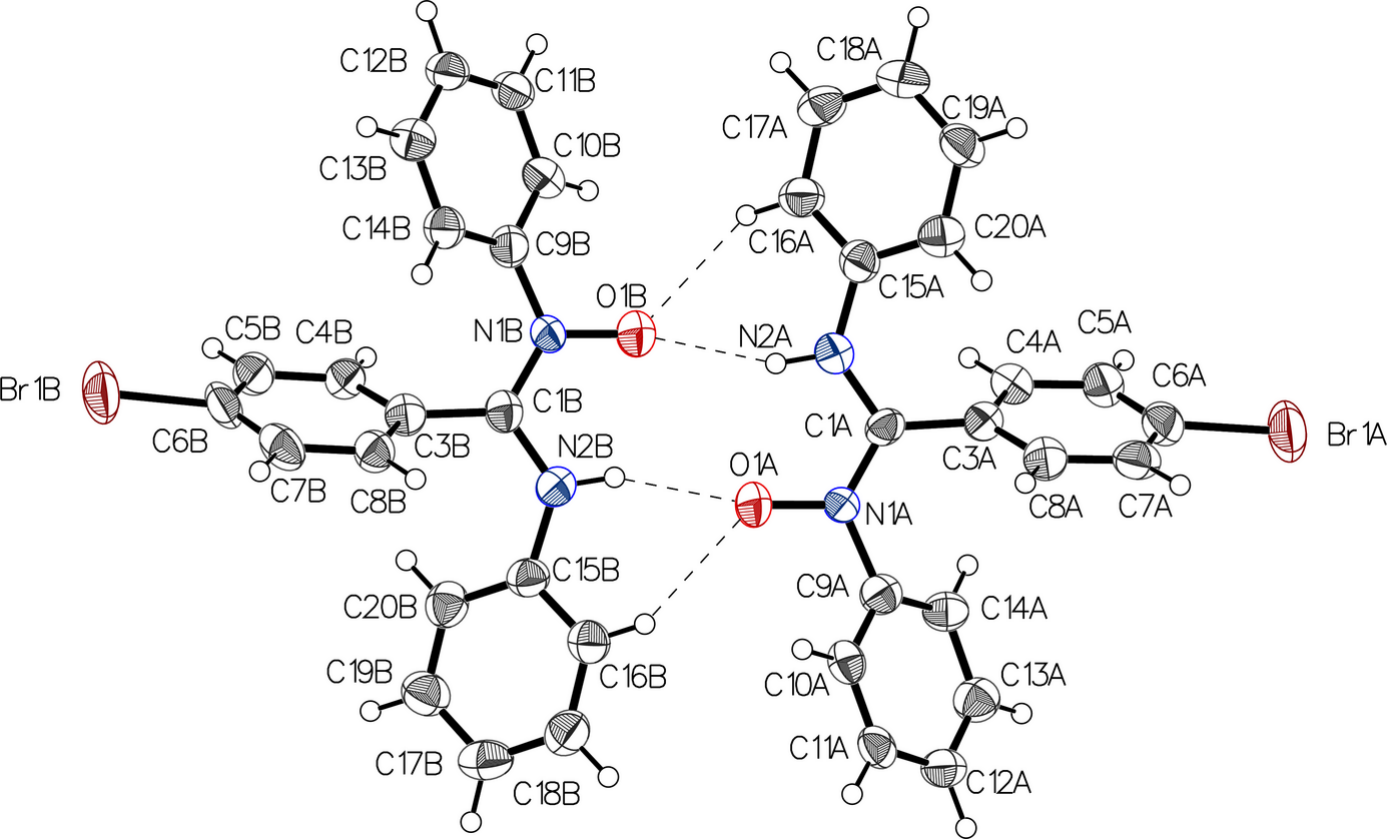
View of the asymmetric unit containing two independent mol­ecules with displacement ellipsoids drawn at the 50% probability level.

The title compound C_19_H_15_BrN_2_O (AMOX-Br) is a symmetrically *N*,*N*′-disubstituted aryl­amidine *N*′-oxide. Its crystal structure was determined as part of a research project involving the synthesis of AMOX ligands and their coordination complexes with trans­ition-metal ions for assessing their photophysical and electrochemical properties (Patel *et al.*, 1979 ▸; Verma *et al.*, 1995 ▸; Cibian *et al.*, 2018 ▸). The asymmetric unit contains two mol­ecules (labelled with suffixes *A* and *B*) in the ortho­rhom­bic *Pna*2_1_ space group. Each mol­ecule contains an O—N—C—N bridge bearing a central *C*-aryl ring with 4-bromo substitution and two side *N*-phenyl rings (Fig. 1 ▸).

**Figure 2:**
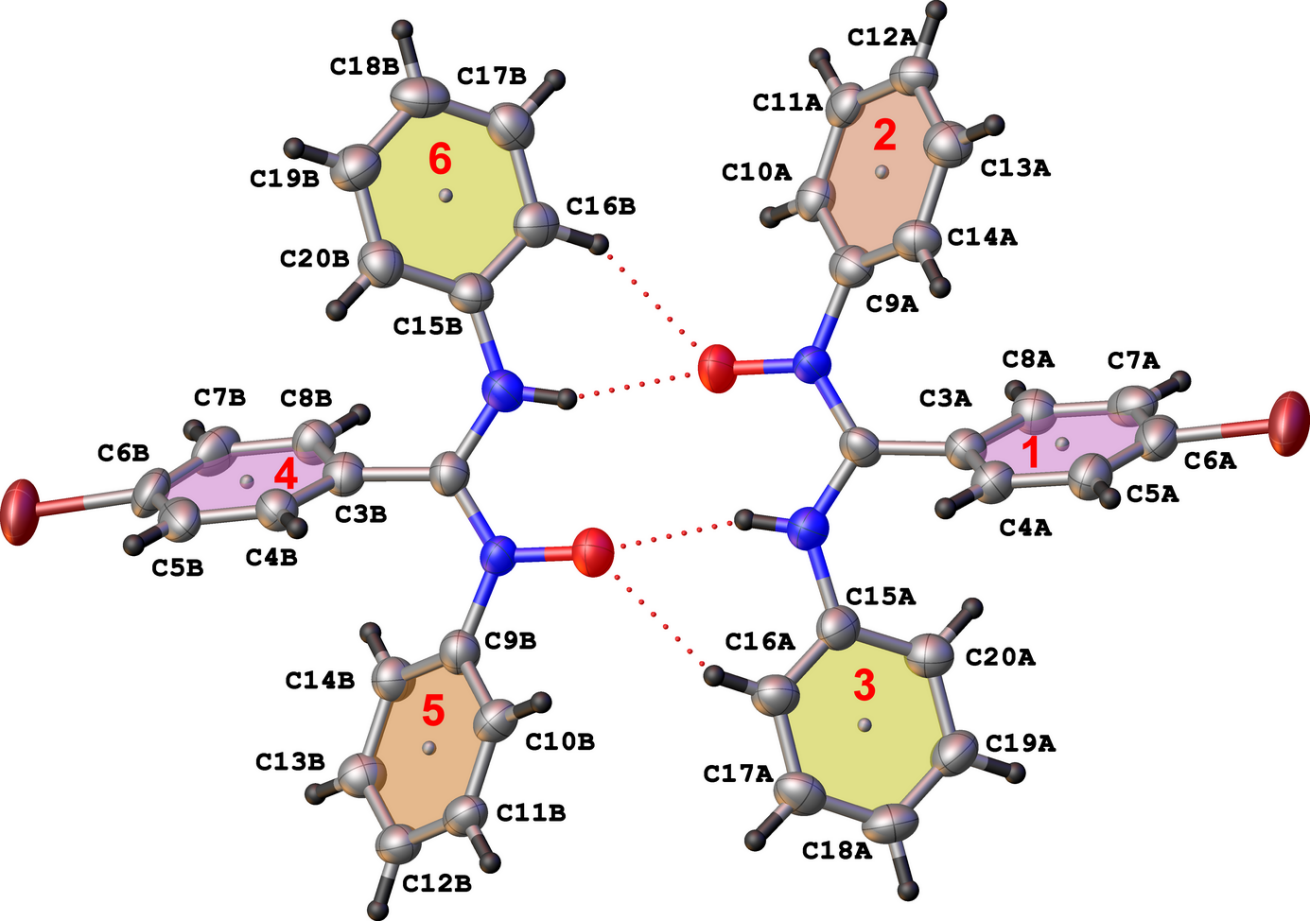
View of planes 1, 2, 3, 4, 5, and 6 for the phenyl rings of the mol­ecules in the asymmetric unit and hydrogen bonds (dotted lines).

The N—C—N moieties in both mol­ecules display electronic delocalization since the bond lengths are shorter than classical C—N single bonds (1.45 Å) and longer than localized C=N double bonds (1.27 Å) (Filgueiras de Athayde-Filho *et al.*, 2003 ▸). This observation is evident from the similar N—C bond distances [C1*A*—N1*A* = 1.321 (11) Å, C1*A*—N2*A* = 1.338 (12) Å and C1*B*—N1*B* = 1.325 (11) Å, C1*B*—N2*B* = 1.366 (11) Å] for the two mol­ecules of the asymmetric unit. Furthermore, these values are comparable to those reported for the N—C—N bond lengths in 4-bromo-*N*-phenyl­benzamidoxime (Cibian *et al.*, 2009 ▸). The N—C—N—O moiety is slightly twisted with a torsion angle of 3.9 (11)° for N2*B*—C1*B*—N1*B*—O1*B* and −3.9 (12)° for O1*A*—N1*A*—C1*A*—N2*A*. Considering the central and side phenyl rings, if rings C3*A*–C8*A*, C9*A*–C14*A* and C15*A*–C20*A* from mol­ecule *A* are labelled 1, 2 and 3, respectively (4, 5 and 6, respectively, for the rings of mol­ecule *B*, Fig. 2 ▸), the angle between planes 1 and 3 is 68.9 (3)° while the angle between planes 1 and 2 is 57.1 (3)°, with the corresponding angles for mol­ecule *B* being 69.6 (3) and 56.9 (3)°, respectively. The angles between the planes of the side *N*-phenyl rings (2/3 & 5/6 for mol­ecules *A* and *B*, respectively) are 73.6 (3) and 72.6 (3)°, respectively.

**Figure 3:**
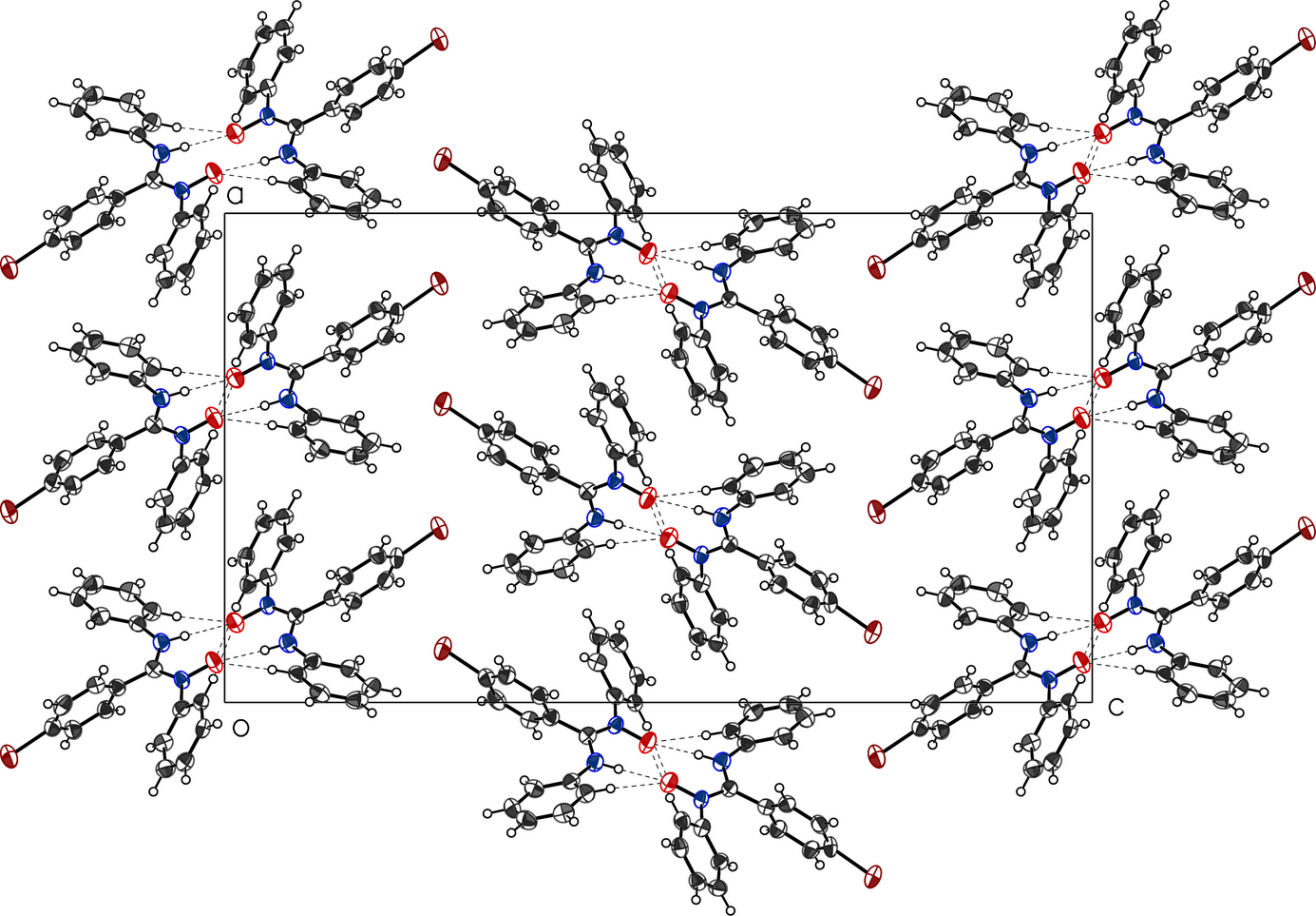
View of the packing of the title compound in the unit cell.

Hydrogen bonds are reported in Table 1 ▸. The extended structure displays cyclic dimers linked by pairs of N—H⋯O and C—H⋯O hydrogen bonds. No notable inter­molecular π–π stacking inter­actions are observed but there are several C—H⋯π contacts that complement the conventional hydrogen bonds (Fig. 3 ▸).

## Synthesis and crystallization

The compound was synthesized with some modification to the procedure reported in the literature (Cibian *et al.*, 2018 ▸): 4-bromo-*N*,*N*′-di­phenyl­benzimidamide, AM–Br (0.84 g, 2.4 mmol, 1.0 eq.) was dissolved in di­chloro­methane in the presence of NaHCO_3_ (1.19 g, 14.2 mmol, 5.0 eq.) and stirred for 10 minutes. Then, *m*-chloro-peroxi­benzoic acid, *m*-CPBA, 70% (0.76 g, 3.1 mmol, 1.1 eq.) was added in small aliquotes to the AM–Br solution. The reaction mixture turned brown instantly and darkened within a minute of stirring. The reaction mixture was stirred at room temperature for 2 h and filtered under vacuum. The filtrate was washed with 2 *M* NaOH solution (2 × 80 ml), distilled water (2 × 80 ml) and brine (3 × 50 ml). The collected di­chloro­methane layer was dried with anhydrous Na_2_SO_4_ and the solvent was removed under vacuum. The sticky brown solid obtained was sonicated in a minimum volume of ethyl acetate/hexa­nes. The precipitate thus formed was filtered and washed with ethyl acetate/hexa­nes to give a pale-yellow amorphous powder (yield 76%) of the title compound. Bright-yellow needles suitable for X-ray diffraction measurements were grown over a period of 5 d at room temperature by the hexane anti-solvent layering technique with a di­chloro­methane solution of the compound (1:1 *v*/*v*).

## Refinement

Crystal data, data collection and structure refinement details are summarized in Table 2 ▸. The crystal studied was refined as a two-component inversion twin.

## Supplementary Material

Crystal structure: contains datablock(s) I. DOI: 10.1107/S2414314624009684/hb4487sup1.cif

Structure factors: contains datablock(s) I. DOI: 10.1107/S2414314624009684/hb4487Isup2.hkl

Supporting information file. DOI: 10.1107/S2414314624009684/hb4487Isup3.cml

CCDC reference: 2388273

Additional supporting information:  crystallographic information; 3D view; checkCIF report

## Figures and Tables

**Table 1:** Hydrogen-bond geometry (Å, °) *Cg*2, *Cg*3, *Cg*5 and *Cg*6 are the centroids of the C9*A*–C14*A*, C15*A*–C20*A*, C9*B*–C14*B* and C15*B*–C20*B* rings, respectively.

*D*—H⋯*A*	*D*—H	H⋯*A*	*D*⋯*A*	*D*—H⋯*A*
N2*A*—H2*A*⋯O1*B*	0.88	2.20	3.005 (11)	152
C10*A*—H10*A*⋯O1*B*^i^	0.95	2.32	3.249 (12)	167
C16*A*—H16*A*⋯O1*B*	0.95	2.41	3.191 (12)	139
N2*B*—H2*B*⋯O1*A*	0.88	2.19	2.999 (10)	152
C10*B*—H10*B*⋯O1*A*^ii^	0.95	2.28	3.214 (12)	167
C16*B*—H16*B*⋯O1*A*	0.95	2.40	3.188 (12)	140
C4*A*—H4*A*⋯*Cg*2^ii^	0.95	2.77	3.614 (10)	149
C4*B*—H4*B*⋯*Cg*6^ii^	0.95	2.64	3.534 (10)	157
C8*A*—H8*A*⋯*Cg*3^i^	0.95	2.71	3.591 (10)	154
C8*B*—H8*B*⋯*Cg*5^i^	0.95	2.85	3.688 (10)	148

Symmetry codes: (i) 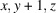; (ii) 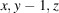.

**Table 2:** Experimental details

Crystal data	
Chemical formula	C_19_H_15_BrN_2_O
*M*_r_	367.24
Crystal system, space group	Orthorhombic, *Pna*2_1_
Temperature (K)	150
*a*, *b*, *c* (Å)	17.7036 (16), 5.8468 (6), 31.418 (3)
*V* (Å^3^)	3252.0 (5)
*Z*	8
Radiation type	Ga *K*α, λ = 1.34139 Å
μ (mm^−1^)	2.30
Crystal size (mm)	0.19 × 0.03 × 0.03

Data collection	
Diffractometer	Bruker Venture Metaljet
Absorption correction	Multi-scan (*SADABS*; Krause *et al.*, 2015 ▸)
*T*_min_, *T*_max_	0.079, 0.201
No. of measured, independent and observed [*I* > 2σ(*I*)] reflections	100504, 6071, 4793
*R*_int_	0.090
(sin θ/λ)_max_ (Å^−1^)	0.610

Refinement	
*R*[*F*^2^ > 2σ(*F*^2^)], *wR*(*F*^2^), *S*	0.055, 0.158, 1.07
No. of reflections	6071
No. of parameters	416
No. of restraints	1
H-atom treatment	H-atom parameters constrained
Δρ_max_, Δρ_min_ (e Å^−3^)	1.13, −0.64
Absolute structure	Refined as an inversion twin.
Absolute structure parameter	0.23 (6)

Computer programs: *APEX4* (Bruker, 2022 ▸), *SAINT* (Bruker, 2020 ▸), *SHELXT* (Sheldrick, 2015*a* ▸), *SHELXL* (Sheldrick, 2015*b* ▸), *OLEX2* (Dolomanov *et al.*, 2009 ▸), *FinalCIF* (Kratzert, 2024 ▸) and *publCIF* (Westrip, 2010 ▸).

## full crystallographic data

### 4-Bromo-N,N'-diphenylbenzimidamide N'-oxide Crystal data

**Table:**

C_19_H_15_BrN_2_O	*D*_x_ = 1.500 Mg m^−^^3^
*M**_r_* = 367.24	Ga *K*α radiation, λ = 1.34139 Å
Orthorhombic, *P**n**a*2_1_	Cell parameters from 9781 reflections
*a* = 17.7036 (16) Å	θ = 4.5–54.4°
*b* = 5.8468 (6) Å	µ = 2.30 mm^−^^1^
*c* = 31.418 (3) Å	*T* = 150 K
*V* = 3252.0 (5) Å^3^	Needle, clear light colourless
*Z* = 8	0.19 × 0.03 × 0.03 mm
*F*(000) = 1488	

### 4-Bromo-N,N'-diphenylbenzimidamide N'-oxide Data collection

**Table:**

Bruker Venture Metaljet diffractometer	6071 independent reflections
Radiation source: Metal Jet, Gallium Liquid Metal Jet Source	4793 reflections with *I* > 2σ(*I*)
Helios MX Mirror Optics monochromator	*R*_int_ = 0.090
Detector resolution: 10.42 pixels mm^-1^	θ_max_ = 54.9°, θ_min_ = 2.5°
ω and φ scans	*h* = −21→21
Absorption correction: multi-scan (SADABS; Krause *et al.*, 2015)	*k* = −6→7
*T*_min_ = 0.079, *T*_max_ = 0.201	*l* = −38→37
100504 measured reflections	

### 4-Bromo-N,N'-diphenylbenzimidamide N'-oxide Refinement

**Table:**

Refinement on *F*^2^	Hydrogen site location: inferred from neighbouring sites
Least-squares matrix: full	H-atom parameters constrained
*R*[*F*^2^ > 2σ(*F*^2^)] = 0.055	*w* = 1/[σ^2^(*F*_o_^2^) + (0.0728*P*)^2^ + 6.1028*P*] where *P* = (*F*_o_^2^ + 2*F*_c_^2^)/3
*wR*(*F*^2^) = 0.158	(Δ/σ)_max_ < 0.001
*S* = 1.07	Δρ_max_ = 1.13 e Å^−^^3^
6071 reflections	Δρ_min_ = −0.64 e Å^−^^3^
416 parameters	Absolute structure: Refined as an inversion twin.
1 restraint	Absolute structure parameter: 0.23 (6)
Primary atom site location: dual	

### 4-Bromo-N,N'-diphenylbenzimidamide N'-oxide Special details

**Table:**

Experimental. X-ray crystallographic data for I were collected from a single crystal sample, which was mounted on a loop fiber. Data were collected using a Bruker Venture diffractometer equipped with a Photon III CMOS Detector, a Helios MX optics and a Kappa goniometer. The crystal-to-detector distance was 4.0 cm, and the data collection was carried out in 1024 x 1024 pixel mode.
Geometry. All esds (except the esd in the dihedral angle between two l.s. planes) are estimated using the full covariance matrix. The cell esds are taken into account individually in the estimation of esds in distances, angles and torsion angles; correlations between esds in cell parameters are only used when they are defined by crystal symmetry. An approximate (isotropic) treatment of cell esds is used for estimating esds involving l.s. planes.
Refinement. Refined as a 2-component inversion twin.Hydrogen atoms attached to nitrogen were first located from Fourier difference map then refined using the equivalent calculated positions. The C-bound H atoms were geometrically placed and refined as riding atoms.

### 4-Bromo-N,N'-diphenylbenzimidamide N'-oxide Fractional atomic coordinates and isotropic or equivalent isotropic displacement parameters (Å^2^)

**Table:**

	*x*	*y*	*z*	*U*_iso_*/*U*_eq_	
Br1A	0.61038 (7)	0.6881 (3)	0.25148 (3)	0.0760 (4)	
O1A	0.4180 (4)	0.7299 (11)	0.4878 (2)	0.0500 (18)	
N1A	0.4538 (4)	0.7506 (11)	0.4507 (3)	0.0344 (17)	
C1A	0.4319 (4)	0.6209 (13)	0.4185 (3)	0.0351 (18)	
N2A	0.3778 (4)	0.4681 (13)	0.4271 (2)	0.0417 (17)	
H2A	0.363970	0.461329	0.453973	0.050*	
C3A	0.4695 (4)	0.6315 (15)	0.3761 (3)	0.0369 (18)	
C4A	0.5143 (5)	0.4453 (15)	0.3630 (3)	0.0402 (19)	
H4A	0.516330	0.310580	0.379883	0.048*	
C5A	0.5558 (5)	0.4585 (18)	0.3253 (3)	0.047 (2)	
H5A	0.586546	0.334943	0.315994	0.057*	
C6A	0.5504 (6)	0.6599 (19)	0.3018 (3)	0.051 (2)	
C7A	0.5056 (6)	0.8449 (17)	0.3136 (3)	0.047 (2)	
H7A	0.503148	0.978161	0.296394	0.057*	
C8A	0.4644 (5)	0.8303 (17)	0.3516 (3)	0.043 (2)	
H8A	0.433335	0.953821	0.360476	0.051*	
C9A	0.5134 (5)	0.9239 (15)	0.4500 (3)	0.0396 (19)	
C10A	0.4990 (5)	1.1212 (15)	0.4729 (3)	0.0380 (19)	
H10A	0.451900	1.142036	0.486832	0.046*	
C11A	0.5530 (5)	1.2838 (15)	0.4752 (3)	0.040 (2)	
H11A	0.543730	1.417141	0.491620	0.048*	
C12A	0.6217 (5)	1.2623 (15)	0.4542 (4)	0.042 (2)	
H12A	0.658205	1.381271	0.455257	0.051*	
C13A	0.6354 (5)	1.0632 (17)	0.4319 (3)	0.048 (2)	
H13A	0.682490	1.045000	0.417789	0.057*	
C14A	0.5821 (5)	0.8882 (14)	0.4296 (3)	0.0409 (19)	
H14A	0.592282	0.750290	0.414638	0.049*	
C15A	0.3389 (5)	0.3147 (16)	0.3995 (3)	0.041 (2)	
C16A	0.3139 (5)	0.1128 (15)	0.4159 (3)	0.044 (2)	
H16A	0.323975	0.076505	0.444787	0.053*	
C17A	0.2739 (5)	−0.0410 (16)	0.3908 (3)	0.047 (2)	
H17A	0.256372	−0.180680	0.402644	0.056*	
C18A	0.2594 (5)	0.0097 (19)	0.3481 (3)	0.052 (3)	
H18A	0.233473	−0.096676	0.330491	0.062*	
C19A	0.2838 (6)	0.2216 (18)	0.3316 (4)	0.051 (3)	
H19A	0.272754	0.260575	0.302911	0.061*	
C20A	0.3238 (5)	0.3733 (16)	0.3568 (3)	0.045 (2)	
H20A	0.340832	0.514948	0.345548	0.055*	
C1B	0.3236 (4)	0.3768 (15)	0.5820 (3)	0.0370 (18)	
Br1B	0.14367 (7)	0.2681 (2)	0.74742 (3)	0.0701 (4)	
N1B	0.3017 (4)	0.2490 (11)	0.5494 (3)	0.0336 (17)	
O1B	0.3366 (4)	0.2703 (11)	0.5123 (2)	0.0478 (17)	
N2B	0.3789 (4)	0.5326 (12)	0.5731 (2)	0.0417 (17)	
H2B	0.393219	0.539951	0.546301	0.050*	
C3B	0.2863 (5)	0.3580 (16)	0.6241 (3)	0.0387 (19)	
C4B	0.2924 (5)	0.1630 (16)	0.6482 (3)	0.0391 (19)	
H4B	0.325401	0.044035	0.639406	0.047*	
C5B	0.2506 (5)	0.1381 (18)	0.6856 (3)	0.048 (2)	
H5B	0.254569	0.001873	0.701917	0.058*	
C6B	0.2046 (6)	0.3074 (19)	0.6984 (3)	0.048 (2)	
C7B	0.1990 (5)	0.5162 (18)	0.6760 (3)	0.050 (2)	
H7B	0.167883	0.637004	0.686058	0.060*	
C8B	0.2411 (5)	0.5390 (16)	0.6382 (3)	0.041 (2)	
H8B	0.238858	0.677153	0.622357	0.049*	
C9B	0.2439 (5)	0.0776 (15)	0.5503 (3)	0.0393 (19)	
C10B	0.2578 (5)	−0.1214 (15)	0.5272 (3)	0.040 (2)	
H10B	0.304907	−0.142167	0.513284	0.049*	
C11B	0.2031 (6)	−0.2875 (16)	0.5248 (3)	0.042 (2)	
H11B	0.212594	−0.422652	0.508851	0.051*	
C12B	0.1340 (5)	−0.2606 (14)	0.5453 (4)	0.041 (2)	
H12B	0.096363	−0.376087	0.543340	0.049*	
C13B	0.1207 (5)	−0.0633 (17)	0.5685 (3)	0.042 (2)	
H13B	0.074096	−0.045649	0.583136	0.051*	
C14B	0.1746 (5)	0.1094 (15)	0.5707 (3)	0.0401 (19)	
H14B	0.164413	0.246787	0.585759	0.048*	
C15B	0.4160 (5)	0.6836 (15)	0.6013 (3)	0.040 (2)	
C16B	0.4421 (4)	0.8874 (15)	0.5840 (3)	0.041 (2)	
H16B	0.433158	0.921877	0.554880	0.050*	
C17B	0.4814 (5)	1.0404 (17)	0.6097 (3)	0.049 (2)	
H17B	0.498950	1.180375	0.597936	0.059*	
C18B	0.4953 (5)	0.9913 (18)	0.6523 (4)	0.054 (3)	
H18B	0.521577	1.097190	0.669808	0.065*	
C19B	0.4695 (6)	0.7814 (18)	0.6691 (5)	0.053 (3)	
H19B	0.479286	0.744164	0.698056	0.063*	
C20B	0.4308 (5)	0.6319 (17)	0.6440 (3)	0.046 (2)	
H20B	0.413583	0.491091	0.655569	0.055*	

### 4-Bromo-N,N'-diphenylbenzimidamide N'-oxide Atomic displacement parameters (Å^2^)

**Table:**

	*U*^11^	*U*^22^	*U*^33^	*U*^12^	*U*^13^	*U*^23^
Br1A	0.0716 (8)	0.1182 (10)	0.0382 (7)	−0.0140 (7)	0.0150 (7)	0.0000 (9)
O1A	0.060 (4)	0.053 (4)	0.038 (4)	−0.010 (3)	0.018 (3)	−0.007 (3)
N1A	0.040 (4)	0.032 (4)	0.031 (5)	−0.006 (3)	0.006 (4)	−0.006 (3)
C1A	0.039 (4)	0.026 (4)	0.040 (5)	−0.002 (3)	0.003 (3)	0.002 (3)
N2A	0.045 (4)	0.044 (4)	0.036 (4)	−0.005 (3)	0.004 (3)	−0.004 (3)
C3A	0.037 (4)	0.036 (4)	0.037 (5)	−0.002 (4)	0.002 (4)	0.000 (4)
C4A	0.042 (5)	0.040 (5)	0.038 (4)	0.000 (4)	−0.001 (4)	−0.004 (4)
C5A	0.048 (5)	0.055 (6)	0.039 (5)	−0.005 (4)	0.003 (4)	−0.004 (4)
C6A	0.046 (5)	0.069 (7)	0.037 (5)	−0.012 (5)	−0.003 (4)	−0.003 (5)
C7A	0.057 (6)	0.040 (5)	0.046 (6)	−0.007 (5)	−0.015 (5)	0.001 (4)
C8A	0.038 (4)	0.047 (5)	0.043 (6)	0.001 (4)	−0.001 (4)	0.001 (4)
C9A	0.038 (4)	0.041 (5)	0.039 (4)	−0.002 (4)	−0.007 (4)	0.005 (4)
C10A	0.039 (4)	0.045 (5)	0.031 (4)	0.004 (4)	−0.002 (3)	0.001 (4)
C11A	0.049 (5)	0.043 (5)	0.028 (5)	0.002 (4)	−0.007 (4)	0.002 (4)
C12A	0.047 (5)	0.036 (5)	0.045 (6)	−0.005 (4)	−0.008 (4)	0.001 (4)
C13A	0.042 (5)	0.051 (6)	0.050 (6)	−0.002 (4)	−0.003 (4)	0.002 (5)
C14A	0.043 (5)	0.032 (4)	0.048 (5)	0.001 (4)	−0.003 (4)	−0.002 (4)
C15A	0.034 (4)	0.050 (5)	0.039 (5)	−0.001 (4)	0.005 (4)	−0.005 (4)
C16A	0.046 (5)	0.039 (5)	0.048 (5)	−0.004 (4)	−0.001 (4)	−0.005 (4)
C17A	0.050 (5)	0.032 (5)	0.059 (6)	−0.003 (4)	0.003 (4)	−0.002 (4)
C18A	0.049 (5)	0.049 (6)	0.057 (6)	−0.008 (5)	−0.010 (4)	−0.012 (5)
C19A	0.052 (6)	0.063 (7)	0.037 (7)	−0.005 (5)	−0.008 (4)	−0.007 (5)
C20A	0.043 (5)	0.045 (5)	0.049 (5)	−0.002 (4)	−0.003 (4)	−0.001 (4)
C1B	0.036 (4)	0.041 (5)	0.034 (4)	0.002 (4)	0.003 (3)	0.000 (4)
Br1B	0.0672 (7)	0.1061 (9)	0.0369 (6)	−0.0024 (6)	0.0184 (6)	0.0002 (7)
N1B	0.041 (4)	0.032 (4)	0.028 (5)	−0.001 (3)	0.004 (3)	−0.004 (3)
O1B	0.053 (4)	0.051 (4)	0.040 (4)	−0.004 (3)	0.010 (3)	−0.002 (3)
N2B	0.046 (4)	0.038 (4)	0.041 (4)	−0.007 (3)	0.006 (3)	−0.001 (3)
C3B	0.033 (4)	0.045 (5)	0.037 (5)	0.001 (4)	−0.001 (3)	0.001 (4)
C4B	0.038 (4)	0.041 (5)	0.038 (5)	−0.003 (4)	0.001 (4)	0.001 (4)
C5B	0.046 (5)	0.061 (6)	0.036 (5)	−0.013 (5)	−0.010 (4)	0.008 (4)
C6B	0.057 (6)	0.063 (6)	0.024 (5)	−0.002 (5)	−0.001 (4)	−0.007 (4)
C7B	0.041 (5)	0.066 (6)	0.043 (5)	0.006 (4)	0.005 (4)	−0.021 (5)
C8B	0.043 (5)	0.039 (5)	0.040 (5)	0.002 (4)	0.004 (4)	−0.002 (4)
C9B	0.043 (5)	0.039 (5)	0.035 (4)	0.000 (4)	0.004 (4)	0.000 (4)
C10B	0.046 (5)	0.040 (5)	0.036 (4)	0.003 (4)	−0.004 (4)	−0.005 (4)
C11B	0.053 (6)	0.037 (5)	0.037 (6)	−0.004 (4)	−0.005 (4)	−0.004 (4)
C12B	0.043 (5)	0.040 (5)	0.040 (6)	−0.005 (4)	−0.005 (4)	−0.002 (4)
C13B	0.036 (4)	0.044 (6)	0.047 (5)	0.003 (4)	−0.003 (4)	−0.003 (4)
C14B	0.037 (4)	0.042 (5)	0.041 (4)	0.001 (4)	0.004 (4)	−0.003 (4)
C15B	0.037 (5)	0.041 (5)	0.042 (5)	−0.005 (4)	−0.003 (4)	0.000 (4)
C16B	0.036 (4)	0.044 (5)	0.044 (5)	0.003 (4)	0.006 (4)	−0.007 (4)
C17B	0.048 (5)	0.044 (5)	0.054 (6)	−0.002 (4)	0.009 (4)	0.001 (5)
C18B	0.042 (5)	0.051 (6)	0.070 (7)	−0.008 (4)	−0.004 (5)	−0.011 (5)
C19B	0.052 (6)	0.057 (7)	0.048 (7)	−0.004 (5)	−0.007 (5)	−0.005 (5)
C20B	0.039 (5)	0.052 (6)	0.046 (5)	−0.001 (4)	0.002 (4)	0.004 (4)

### 4-Bromo-N,N'-diphenylbenzimidamide N'-oxide Geometric parameters (Å, º)

**Table:**

Br1A—C6A	1.911 (10)	C1B—N1B	1.325 (11)
O1A—N1A	1.333 (11)	C1B—N2B	1.366 (11)
N1A—C1A	1.321 (11)	C1B—C3B	1.485 (12)
N1A—C9A	1.463 (11)	Br1B—C6B	1.894 (10)
C1A—N2A	1.338 (11)	N1B—O1B	1.326 (10)
C1A—C3A	1.491 (12)	N1B—C9B	1.433 (11)
N2A—H2A	0.8800	N2B—H2B	0.8800
N2A—C15A	1.423 (12)	N2B—C15B	1.415 (12)
C3A—C4A	1.408 (12)	C3B—C4B	1.373 (14)
C3A—C8A	1.398 (14)	C3B—C8B	1.398 (13)
C4A—H4A	0.9500	C4B—H4B	0.9500
C4A—C5A	1.397 (13)	C4B—C5B	1.396 (13)
C5A—H5A	0.9500	C5B—H5B	0.9500
C5A—C6A	1.393 (15)	C5B—C6B	1.343 (15)
C6A—C7A	1.392 (15)	C6B—C7B	1.413 (15)
C7A—H7A	0.9500	C7B—H7B	0.9500
C7A—C8A	1.400 (14)	C7B—C8B	1.407 (13)
C8A—H8A	0.9500	C8B—H8B	0.9500
C9A—C10A	1.383 (12)	C9B—C10B	1.393 (12)
C9A—C14A	1.391 (12)	C9B—C14B	1.396 (12)
C10A—H10A	0.9500	C10B—H10B	0.9500
C10A—C11A	1.350 (13)	C10B—C11B	1.374 (13)
C11A—H11A	0.9500	C11B—H11B	0.9500
C11A—C12A	1.387 (14)	C11B—C12B	1.392 (14)
C12A—H12A	0.9500	C12B—H12B	0.9500
C12A—C13A	1.381 (14)	C12B—C13B	1.385 (14)
C13A—H13A	0.9500	C13B—H13B	0.9500
C13A—C14A	1.394 (13)	C13B—C14B	1.391 (13)
C14A—H14A	0.9500	C14B—H14B	0.9500
C15A—C16A	1.361 (13)	C15B—C16B	1.389 (13)
C15A—C20A	1.410 (13)	C15B—C20B	1.398 (13)
C16A—H16A	0.9500	C16B—H16B	0.9500
C16A—C17A	1.391 (13)	C16B—C17B	1.391 (13)
C17A—H17A	0.9500	C17B—H17B	0.9500
C17A—C18A	1.397 (15)	C17B—C18B	1.393 (15)
C18A—H18A	0.9500	C18B—H18B	0.9500
C18A—C19A	1.412 (16)	C18B—C19B	1.411 (15)
C19A—H19A	0.9500	C19B—H19B	0.9500
C19A—C20A	1.384 (14)	C19B—C20B	1.364 (15)
C20A—H20A	0.9500	C20B—H20B	0.9500
			
O1A—N1A—C9A	114.7 (7)	N1B—C1B—N2B	115.3 (7)
C1A—N1A—O1A	118.6 (7)	N1B—C1B—C3B	121.2 (8)
C1A—N1A—C9A	126.7 (8)	N2B—C1B—C3B	123.4 (7)
N1A—C1A—N2A	116.1 (7)	C1B—N1B—O1B	119.3 (7)
N1A—C1A—C3A	121.9 (7)	C1B—N1B—C9B	126.0 (8)
N2A—C1A—C3A	121.8 (7)	O1B—N1B—C9B	114.6 (7)
C1A—N2A—H2A	114.9	C1B—N2B—H2B	115.8
C1A—N2A—C15A	130.1 (8)	C1B—N2B—C15B	128.4 (7)
C15A—N2A—H2A	114.9	C15B—N2B—H2B	115.8
C4A—C3A—C1A	118.7 (8)	C4B—C3B—C1B	121.2 (8)
C8A—C3A—C1A	119.9 (8)	C4B—C3B—C8B	119.9 (9)
C8A—C3A—C4A	121.2 (9)	C8B—C3B—C1B	118.8 (8)
C3A—C4A—H4A	120.0	C3B—C4B—H4B	119.7
C5A—C4A—C3A	120.1 (9)	C3B—C4B—C5B	120.6 (9)
C5A—C4A—H4A	120.0	C5B—C4B—H4B	119.7
C4A—C5A—H5A	121.3	C4B—C5B—H5B	120.1
C6A—C5A—C4A	117.4 (9)	C6B—C5B—C4B	119.8 (9)
C6A—C5A—H5A	121.3	C6B—C5B—H5B	120.1
C5A—C6A—Br1A	118.2 (8)	C5B—C6B—Br1B	119.9 (8)
C5A—C6A—C7A	123.7 (10)	C5B—C6B—C7B	122.0 (9)
C7A—C6A—Br1A	118.1 (8)	C7B—C6B—Br1B	118.0 (7)
C6A—C7A—H7A	120.7	C6B—C7B—H7B	121.2
C6A—C7A—C8A	118.5 (9)	C8B—C7B—C6B	117.7 (9)
C8A—C7A—H7A	120.7	C8B—C7B—H7B	121.2
C3A—C8A—C7A	119.1 (9)	C3B—C8B—C7B	119.9 (9)
C3A—C8A—H8A	120.4	C3B—C8B—H8B	120.1
C7A—C8A—H8A	120.4	C7B—C8B—H8B	120.1
C10A—C9A—N1A	116.0 (7)	C10B—C9B—N1B	116.6 (7)
C10A—C9A—C14A	121.7 (8)	C10B—C9B—C14B	120.3 (8)
C14A—C9A—N1A	122.2 (8)	C14B—C9B—N1B	122.9 (8)
C9A—C10A—H10A	120.5	C9B—C10B—H10B	120.1
C11A—C10A—C9A	119.0 (8)	C11B—C10B—C9B	119.7 (8)
C11A—C10A—H10A	120.5	C11B—C10B—H10B	120.1
C10A—C11A—H11A	118.9	C10B—C11B—H11B	119.5
C10A—C11A—C12A	122.1 (9)	C10B—C11B—C12B	120.9 (9)
C12A—C11A—H11A	118.9	C12B—C11B—H11B	119.5
C11A—C12A—H12A	120.9	C11B—C12B—H12B	120.4
C13A—C12A—C11A	118.1 (9)	C13B—C12B—C11B	119.1 (8)
C13A—C12A—H12A	120.9	C13B—C12B—H12B	120.4
C12A—C13A—H13A	119.1	C12B—C13B—H13B	119.5
C12A—C13A—C14A	121.8 (9)	C12B—C13B—C14B	120.9 (9)
C14A—C13A—H13A	119.1	C14B—C13B—H13B	119.5
C9A—C14A—C13A	117.2 (8)	C9B—C14B—H14B	120.5
C9A—C14A—H14A	121.4	C13B—C14B—C9B	118.9 (8)
C13A—C14A—H14A	121.4	C13B—C14B—H14B	120.5
C16A—C15A—N2A	118.3 (9)	C16B—C15B—N2B	116.3 (8)
C16A—C15A—C20A	120.5 (9)	C16B—C15B—C20B	120.0 (9)
C20A—C15A—N2A	121.2 (8)	C20B—C15B—N2B	123.6 (8)
C15A—C16A—H16A	119.6	C15B—C16B—H16B	120.3
C15A—C16A—C17A	120.9 (9)	C15B—C16B—C17B	119.4 (9)
C17A—C16A—H16A	119.6	C17B—C16B—H16B	120.3
C16A—C17A—H17A	120.0	C16B—C17B—H17B	119.5
C16A—C17A—C18A	120.1 (9)	C16B—C17B—C18B	120.9 (9)
C18A—C17A—H17A	120.0	C18B—C17B—H17B	119.5
C17A—C18A—H18A	120.6	C17B—C18B—H18B	120.6
C17A—C18A—C19A	118.8 (9)	C17B—C18B—C19B	118.9 (10)
C19A—C18A—H18A	120.6	C19B—C18B—H18B	120.6
C18A—C19A—H19A	119.7	C18B—C19B—H19B	119.9
C20A—C19A—C18A	120.6 (11)	C20B—C19B—C18B	120.2 (12)
C20A—C19A—H19A	119.7	C20B—C19B—H19B	119.9
C15A—C20A—H20A	120.4	C15B—C20B—H20B	119.7
C19A—C20A—C15A	119.1 (10)	C19B—C20B—C15B	120.7 (10)
C19A—C20A—H20A	120.4	C19B—C20B—H20B	119.7
			
Br1A—C6A—C7A—C8A	−176.9 (7)	C1B—N1B—C9B—C10B	−140.5 (9)
O1A—N1A—C1A—N2A	−3.9 (12)	C1B—N1B—C9B—C14B	43.6 (13)
O1A—N1A—C1A—C3A	−179.3 (8)	C1B—N2B—C15B—C16B	−151.1 (8)
O1A—N1A—C9A—C10A	−37.2 (11)	C1B—N2B—C15B—C20B	32.5 (14)
O1A—N1A—C9A—C14A	139.3 (8)	C1B—C3B—C4B—C5B	−173.1 (8)
N1A—C1A—N2A—C15A	175.7 (8)	C1B—C3B—C8B—C7B	173.5 (8)
N1A—C1A—C3A—C4A	109.5 (9)	Br1B—C6B—C7B—C8B	−176.4 (7)
N1A—C1A—C3A—C8A	−65.9 (11)	N1B—C1B—N2B—C15B	−176.6 (8)
N1A—C9A—C10A—C11A	177.1 (8)	N1B—C1B—C3B—C4B	66.9 (11)
N1A—C9A—C14A—C13A	−178.4 (8)	N1B—C1B—C3B—C8B	−109.9 (10)
C1A—N1A—C9A—C10A	141.1 (9)	N1B—C9B—C10B—C11B	−176.4 (8)
C1A—N1A—C9A—C14A	−42.3 (13)	N1B—C9B—C14B—C13B	177.5 (8)
C1A—N2A—C15A—C16A	149.9 (9)	O1B—N1B—C9B—C10B	37.1 (11)
C1A—N2A—C15A—C20A	−32.7 (14)	O1B—N1B—C9B—C14B	−138.8 (8)
C1A—C3A—C4A—C5A	−174.1 (8)	N2B—C1B—N1B—O1B	3.9 (11)
C1A—C3A—C8A—C7A	174.2 (8)	N2B—C1B—N1B—C9B	−178.6 (8)
N2A—C1A—C3A—C4A	−65.7 (11)	N2B—C1B—C3B—C4B	−117.0 (10)
N2A—C1A—C3A—C8A	119.0 (9)	N2B—C1B—C3B—C8B	66.2 (11)
N2A—C15A—C16A—C17A	178.3 (8)	N2B—C15B—C16B—C17B	−177.9 (8)
N2A—C15A—C20A—C19A	−178.0 (9)	N2B—C15B—C20B—C19B	177.4 (9)
C3A—C1A—N2A—C15A	−8.9 (14)	C3B—C1B—N1B—O1B	−179.8 (7)
C3A—C4A—C5A—C6A	−0.3 (13)	C3B—C1B—N1B—C9B	−2.3 (13)
C4A—C3A—C8A—C7A	−1.0 (13)	C3B—C1B—N2B—C15B	7.1 (14)
C4A—C5A—C6A—Br1A	177.0 (7)	C3B—C4B—C5B—C6B	−0.8 (14)
C4A—C5A—C6A—C7A	−0.8 (14)	C4B—C3B—C8B—C7B	−3.3 (13)
C5A—C6A—C7A—C8A	0.9 (14)	C4B—C5B—C6B—Br1B	176.7 (7)
C6A—C7A—C8A—C3A	0.0 (13)	C4B—C5B—C6B—C7B	−2.4 (14)
C8A—C3A—C4A—C5A	1.2 (13)	C5B—C6B—C7B—C8B	2.7 (14)
C9A—N1A—C1A—N2A	177.8 (8)	C6B—C7B—C8B—C3B	0.2 (13)
C9A—N1A—C1A—C3A	2.4 (13)	C8B—C3B—C4B—C5B	3.7 (13)
C9A—C10A—C11A—C12A	1.9 (14)	C9B—C10B—C11B—C12B	−0.4 (15)
C10A—C9A—C14A—C13A	−2.0 (13)	C10B—C9B—C14B—C13B	1.7 (13)
C10A—C11A—C12A—C13A	−2.6 (15)	C10B—C11B—C12B—C13B	−0.2 (16)
C11A—C12A—C13A—C14A	0.9 (15)	C11B—C12B—C13B—C14B	1.6 (15)
C12A—C13A—C14A—C9A	1.3 (14)	C12B—C13B—C14B—C9B	−2.4 (14)
C14A—C9A—C10A—C11A	0.5 (13)	C14B—C9B—C10B—C11B	−0.3 (13)
C15A—C16A—C17A—C18A	0.6 (14)	C15B—C16B—C17B—C18B	0.4 (13)
C16A—C15A—C20A—C19A	−0.6 (14)	C16B—C15B—C20B—C19B	1.1 (14)
C16A—C17A—C18A—C19A	−2.1 (14)	C16B—C17B—C18B—C19B	0.8 (14)
C17A—C18A—C19A—C20A	2.2 (15)	C17B—C18B—C19B—C20B	−1.0 (15)
C18A—C19A—C20A—C15A	−0.9 (15)	C18B—C19B—C20B—C15B	0.1 (15)
C20A—C15A—C16A—C17A	0.8 (14)	C20B—C15B—C16B—C17B	−1.3 (13)

### 4-Bromo-N,N'-diphenylbenzimidamide N'-oxide Hydrogen-bond geometry (Å, º)

*Cg*2, *Cg*3, *Cg*5 and *Cg*6 are the centroids of the C9*A*–C14*A*, C15*A*–C20*A*, C9*B*–C14*B* and C15*B*–C20*B* rings, respectively.

**Table:**

*D*—H···*A*	*D*—H	H···*A*	*D*···*A*	*D*—H···*A*
N2*A*—H2*A*···O1*B*	0.88	2.20	3.005 (11)	152
C10*A*—H10*A*···O1*B*^i^	0.95	2.32	3.249 (12)	167
C16*A*—H16*A*···O1*B*	0.95	2.41	3.191 (12)	139
N2*B*—H2*B*···O1*A*	0.88	2.19	2.999 (10)	152
C10*B*—H10*B*···O1*A*^ii^	0.95	2.28	3.214 (12)	167
C16*B*—H16*B*···O1*A*	0.95	2.40	3.188 (12)	140
C4*A*—H4*A*···*Cg*2^ii^	0.95	2.77	3.614 (10)	149
C4*B*—H4*B*···*Cg*6^ii^	0.95	2.64	3.534 (10)	157
C8*A*—H8*A*···*Cg*3^i^	0.95	2.71	3.591 (10)	154
C8*B*—H8*B*···*Cg*5^i^	0.95	2.85	3.688 (10)	148

Symmetry codes: (i) *x*, *y*+1, *z*; (ii) *x*, *y*−1, *z*.
